# An anthropomorphic phantom for atrial transseptal puncture simulation training

**DOI:** 10.1186/s41205-024-00241-y

**Published:** 2024-10-30

**Authors:** Aya Mutaz Zeidan, Zhouyang Xu, Lisa Leung, Calum Byrne, Sachin Sabu, Yijia Zhou, Christopher Aldo Rinaldi, John Whitaker, Steven E. Williams, Jonathan Behar, Aruna Arujuna, R. James Housden, Kawal Rhode

**Affiliations:** 1 Department of Surgical & Interventional Engineering, King’s College London, London, SE1 7EH UK; 2 Cardiology Department, Guy’s & St Thomas’ NHS Foundation Trust, London, SE1 7EH UK; 3 St. George’s Hospital, NHS Foundation Trust, London, SW17 0QT UK; 4 Center for Cardiovascular Science, University of Edinburgh, Edinburgh, UK

**Keywords:** 3D printing, Transseptal puncture, Training, Simulation, Patient-specific, Cardiology

## Abstract

**Background:**

Transseptal puncture (TSP) is a critical prerequisite for left-sided cardiac interventions, such as atrial fibrillation (AF) ablation and left atrial appendage closure. Despite its routine nature, TSP can be technically demanding and carries a risk of complications. This study presents a novel, patient-specific, anthropomorphic phantom for TSP simulation training that can be used with X-ray fluoroscopy and ultrasound imaging.

**Methods:**

The TSP phantom was developed using additive manufacturing techniques and features a replaceable fossa ovalis (FO) component to allow for multiple punctures without replacing the entire model. Four cardiologists and one cardiology trainee performed TSP on the simulator, and their performance was assessed using four metrics: global isotropy index, distance from the centroid, time taken to perform TSP, and a set of 5-point Likert scale questions to evaluate the clinicians’ perception of the phantom’s realism and utility.

**Results:**

The results demonstrate the simulator’s potential as a training tool for interventional cardiology, providing a realistic and controllable environment for clinicians to refine their TSP skills. Experienced cardiologists tended to cluster their puncture points closer to regions of the FO associated with higher global isotropy index scores, indicating a relationship between experience and optimal puncture localization. The questionnaire analysis revealed that participants generally agreed on the phantom’s realistic anatomical representation and ability to accurately visualize the TSP site under fluoroscopic guidance.

**Conclusions:**

The TSP simulator can be incorporated into training programs, offering trainees the opportunity to improve tool handling, spatial coordination, and manual dexterity prior to performing the procedure on patients. Further studies with larger sample sizes and longitudinal assessments are needed to establish the simulator’s impact on TSP performance and patient outcomes.

## Background

Atrial fibrillation (AF) is the most prevalent cardiac arrhythmia, with epidemiological projections forecasted to affect up to 17.9 million people in Europe alone by 2060 [1, 2]. As the number of AF cases continues to rise, the demand for left-sided cardiac interventions, such as catheter ablation, is expected to grow accordingly. The success of these procedures relies heavily on the operator’s experience in performing transseptal puncture (TSP), a critical preprocedural step in accessing the left atrium (LA) [3, 4]. TSP involves navigating a needle through the fossa ovalis (FO) with precision and accuracy to minimize the risk of complications, such as cardiac perforation and tamponade [4, 5].

Trainees currently conduct TSP on live patients under the careful monitoring of experienced mentors. However, this training approach is associated with higher failure rates, a steep learning curve, increased procedural times, and a greater risk of complications than procedures performed by skilled operators [4, 6–8]. Although trainees can review X-ray fluoroscopy and ultrasound images before live patient procedures, these resources do not provide the haptic feedback necessary for developing the required dexterity, experience with multi-tool manipulation, spatial coordination, and decision-making skills. Experienced operators rely on their ability to interpret the tactile feedback from the proximal end of the TSP kit as it contacts the interatrial septum (IAS) and FO, a skill that is challenging to acquire without hands-on experience [9].

To address these challenges, there is a pressing need for realistic TSP simulators that can provide trainees with the opportunity to practice and refine their skills in a risk-free environment. Morais et al. conducted a systematic review of simulation-based training in TSP, emphasizing the importance of high-fidelity simulators that provide anatomical and procedural experiences [10]. The review highlighted the need for simulators that accurately mimic the mechanical properties of cardiac tissues to enhance their realism and educational value, a point further underscored by [11–13].

Simulation-based medical training has gained recognition for providing a safe environment for clinicians to train and refine their skills without compromising patient safety [3]. Virtual reality simulators have shown promise in shortening training times and improving post-training performance [3]. However, existing TSP models often lack compatibility with the imaging modalities required for guiding TSP and are instead simulated, namely X-ray fluoroscopy and ultrasound, including transesophageal echocardiography (TOE) and intracardiac echocardiography (ICE) [12, 14–16]. The ability to simulate procedural image guidance is essential for providing realistic training experiences. The demand for training models that accurately replicate these conditions has been highlighted by several studies [4, 17].

Recent advancements in TSP simulators have focused on improving anatomical accuracy, haptic feedback, and imaging capabilities. Bezek et al. (2020) developed a 3D-printed TSP model using tissue-mimicking materials and evaluated its performance through mechanical testing and user feedback [12]. While their simulator provided a high degree of anatomical accuracy and customizability, it lacked advanced imaging capabilities and required expensive 3D printing equipment. Thompson et al. (2021, 2023) developed a soft robotic TSP simulator with an ICE environment that provides live visual feedback of the FO [14, 15]. Their simulated ICE subsystem used a depth camera to detect FO deformation and produce real-time simulated ultrasound images. This setup presented complexity and high maintenance requirements.

Zimmermann et al. (2020, 2021) introduced an augmented physical simulator for transcatheter cardiovascular interventions, including TSP [18, 19]. The simulator was evaluated using qualitative physician assessments of haptic feedback and realism. While it provided real-time 3D guidance and high anatomical accuracy, the complex setup may limit its widespread adoption. James et al. (2020) compared virtual reality-guided and fluoroscopy-guided TSP in a cardiac phantom, assessing user performance metrics and gathering feedback [16]. Although their VR system offered an interactive and safe training environment with real-time feedback, it required technical expertise to set up and maintain.

These works often demand additional setups not typically required in real procedures. Moreover, while these simulators addressed haptic realism, they were evaluated subjectively through content validation questionnaire analyses and lacked biomechanical values comparable to human tissue properties reported in the literature [11, 20]. The evaluation methods used to assess TSP simulators can be summarized as follows: (1) user feedback and qualitative assessments relying on the subjective opinions of experienced clinicians to evaluate the realism and training utility of the simulators [15, 18, 19]; (2) mechanical testing on the materials used in the simulators to compare their properties with those of human cardiac tissues [11, 12]; and (3) evaluation of the impact of simulator training on user performance metrics, such as procedural time and success rates [3].

Commercial phantoms, such as the HeartRoid (Heartroid$^{\circledR }$, JMC Corporation, Kanagawa, Japan), and endovascular simulators, like the ANGIO Mentor (Surgical Science, Göteborg, Sweden), are also available [21]. However, these are often expensive, which can be a significant barrier for many institutions and training programs [22]. Moreover, the methods and materials used to develop commercial phantoms are often closed-source and proprietary, making it difficult for researchers and educators to objectively assess their fidelity and accuracy in replicating human anatomy and tissue properties.

The development of anatomically accurate and functional TSP phantoms has primarily involved mold casting and additive manufacturing (AM) techniques [10, 12, 14, 16]. Silicone rubbers and polyvinyl alcohol cryogel are the most commonly used materials in mold casting [10, 14, 15, 18, 19]. At the same time, material jetting polymers such as TangoPlus (PolyJet Objet500, Stratasys, Eden Prairie, Minnesota, U.S.A.) are preferred in AM for their ability to replicate fine anatomical details [12, 23]. However, this relies on the Stratasys Polyjet Material Jetting (MJ) 3D printer (PolyJet Objet500, Stratasys, Eden Prairie, Minnesota, U.S.A.), which can cost upwards of $330,000. Material extrusion (commonly known as fused deposition modeling (FDM)) is another popular manufacturing technique offering improved cost alternatives and various materials [23]. Although these simulators have demonstrated the ability to recreate patient-specific cardiac anatomy, quantitative data on the force and haptic feedback provided by the IAS and FO are scarce [11]. Only one study has conducted direct tensile testing of the FO materials for comparison against human tissue properties [12], highlighting the need to identify materials that closely resemble the biomechanical properties of the human FO [11, 20].

Despite the advancements made by these simulators, several limitations motivate the development of an improved TSP simulator. Firstly, many existing simulators lack the ability to achieve a realistic appearance under X-ray fluoroscopy or ultrasound imaging, which is essential for guiding TSP procedures. Secondly, the materials used in some simulators may not accurately mimic the mechanical properties of human cardiac tissue, limiting the realism of the haptic feedback experienced by trainees. Finally, the high costs associated with advanced manufacturing techniques and complex setups may hinder the widespread adoption of these simulators in training programs.

The primary objective of this study was to develop and evaluate a novel, patient-specific, anthropomorphic phantom for TSP simulation training that addresses these limitations. The proposed phantom aimed to fulfill the following key requirements:

1. **Anatomical accuracy**: The phantom should provide a realistic representation of patient-specific cardiac anatomy, particularly the FO and IAS, to ensure a high-fidelity training experience.
2. **Imaging capability**: The phantom should be capable of being imaged with X-ray fluoroscopy and ultrasound modalities, enabling trainees to practice TSP under various guidance scenarios that closely resemble real-world clinical settings.
3. **Replaceable FO component**: The phantom should feature a replaceable FO component to allow for multiple punctures without replacing the entire model, improving cost-effectiveness and sustainability in clinical training programs.
4. **Quantitative performance assessment**: The phantom should incorporate quantitative metrics, such as the global isotropy index (GII) introduced by [24], to provide objective feedback on TSP performance, facilitating the development of optimal puncture localization and catheter maneuverability skills.
5. **User feedback**: Clinicians’ perception of the realism and utility of the phantom as a training tool should be assessed.
6. **Ease of use**: The phantom should be user-friendly and require minimal technical expertise to set up and operate.

By addressing these key requirements, our proposed TSP simulator aims to provide a comprehensive and accessible training platform that can effectively prepare trainees for the challenges of real-world TSP procedures in a risk-free environment, ultimately improving patient outcomes and safety.

## Methods

### Phantom fabrication

**Fig. 1:**
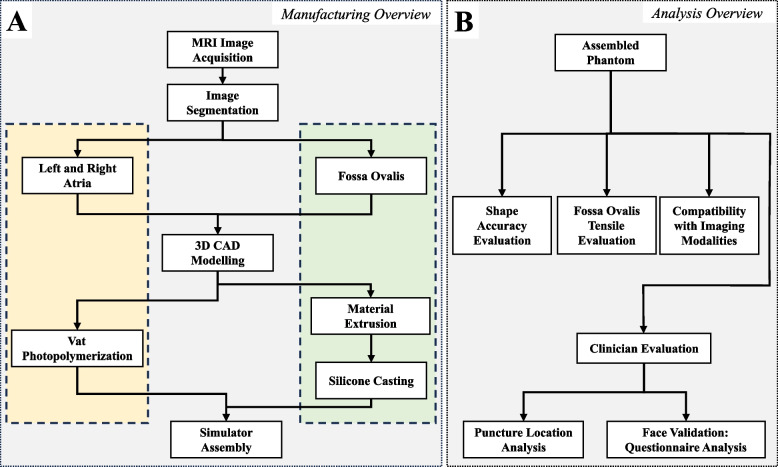
Phantom manufacturing and analysis overview of a patient-specific TSP simulator. Yellow: biatrial structure. Green: Replaceable FO. Abbreviations: TSP (transseptal puncture); MRI (magnetic resonance imaging); CAD (computer-aided design); FO (fossa ovalis)

The overall manufacturing process for the anthropomorphic biatrial phantom is illustrated in Fig. 1. Three additive manufacturing processes were employed: material extrusion (Original Prusa MK4, Prusa Research, Prague, Czech Republic), vat photopolymerization (commonly referred to as stereolithography (SLA)) (Form 3+, Formlabs, Somerville, MA, U.S.A.), and silicone molding (EcoFlex$^{TM}$ & Slacker$^{TM}$, Smooth-On Inc., Macungie, PA, U.S.A.). The three-dimensional models developed in this study, encompassing anatomically precise representations of the LA, right atrium (RA), and FO insert, have been made publicly accessible as standard tessellation language (STL) files via GitHub [25]. This open-source dissemination of the models aims to enhance transparency and facilitate future research endeavors and simulation-based training initiatives in the field.

### MRI data acquisition and reconstruction

**Fig. 2:**
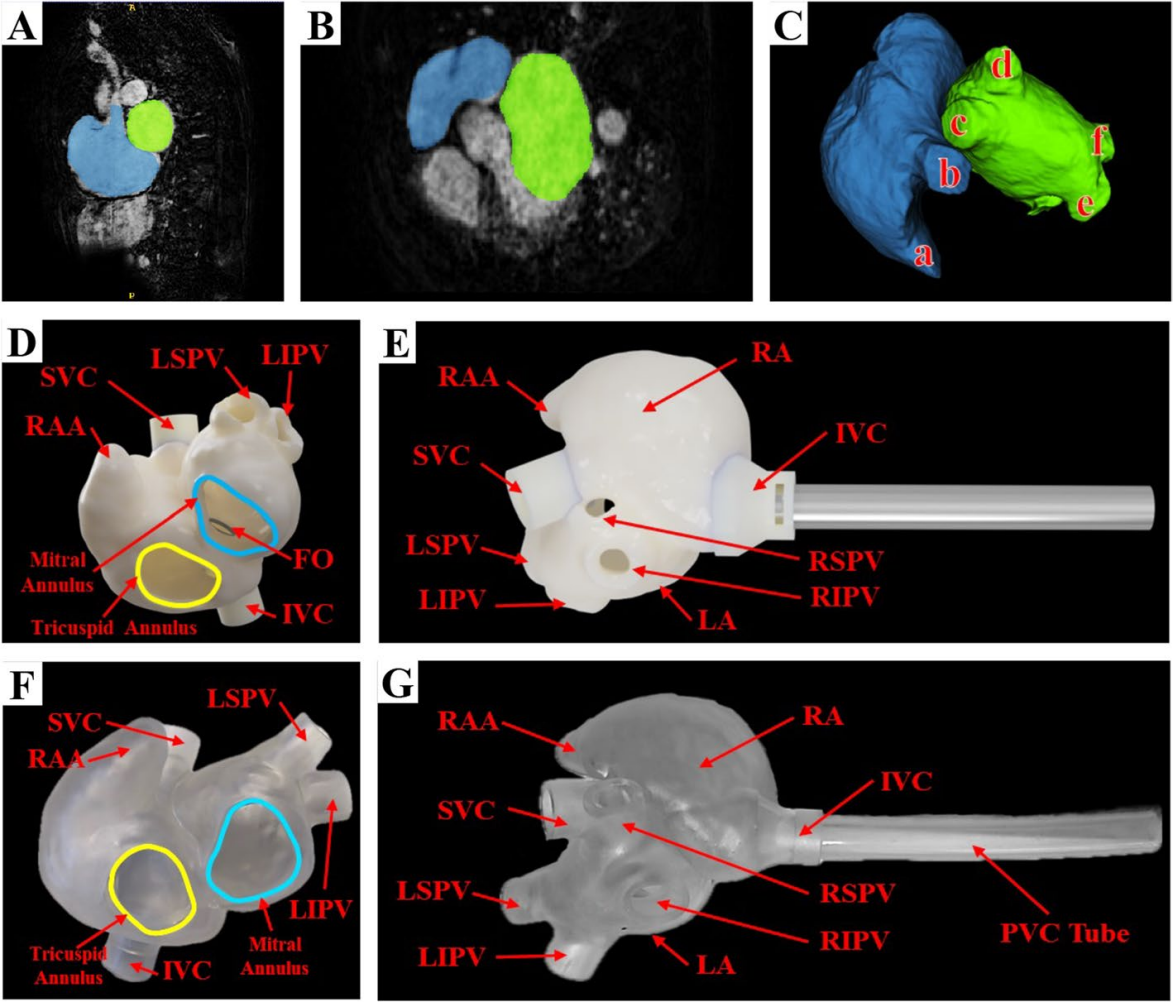
Mapping the segmentation of the LA (green) and RA (blue) based on a cardiac MRI scan of an AF patient. **A** Sagittal view. **B** Transverse view. **C** 3D visualization, including: (a) RAA, (b) SVC, (c) RSPV, (d) RIPV, (e) LSPV, and (f) LIPV. **D, E** and **F, G** visualize CAD renderings and images of the fabricated phantom in two oblique views. Abbreviations: RA (right atrium); LA (left atrium); MRI (magnetic resonance imaging); AF (atrial fibrillation); RAA (right atrial appendage); SVC (superior vena cava), IVC (inferior vena cava); RSPV (right superior pulmonary vein); RIPV (right inferior pulmonary vein); LSPV (left superior pulmonary vein); LIPV (left inferior pulmonary vein); CAD (computer-aided design); FO (fossa ovalis); PVC (polyvinyl chloride)

Cardiac magnetic resonance imaging (MRI) data were acquired from an AF patient who had not previously undergone catheter ablation. The MRI scans were performed on a routine clinical 1.5T scanner (Philips Achieva, v3.2.2, Philips Healthcare, Best, The Netherlands) with a voxel resolution of 0.89 x 0.89 x 1.0 mm$^{3}$. The image represented a single cardiac phase 3D volume acquired during a balanced steady-state free precession sequence with a free-breathing respiratory navigator at end-expiration. The left and right atria were segmented in the image data using a fully automated approach based on a statistical shape model (visualized in Fig. 2A-C) from Philips (Philips, Amsterdam, The Netherlands) [26]. The LA, RA, inferior vena cava (IVC), superior vena cava (SVC), IAS, and FO were then manually delineated from the blood pool. An expert clinician manually traced the FO ($FO~circularity = 1.0$ [20]) from the IAS segmentation and reviewed the biatrial structure segmentation to confirm the accuracy of the anatomical representations. A surface mesh file was then computed from the segmentation (Fig. 2D, E).

### 3D computer-aided design modeling

#### Left and right atria

The ventricular segments were intentionally omitted from the model to enhance visualization within the atrial chambers and to allow access to the FO for replacement after each puncture. This design decision facilitated the creation of an entryway into the atria, allowing clinicians to monitor the FO puncture process with or without imaging modalities. Following the LA and RA segmentation, they were modeled using computer-aided design (CAD) software (Fusion 360, Autodesk, San Francisco, CA, U.S.A.), where approximately 3.75 mm was extruded from the blood pool to create the cardiac tissue shell, more than defined in the literature, to preserve model integrity [12].

The atria were combined into a unified structure using Meshmixer (Autodesk, San Francisco, CA, U.S.A.). The mesh was imported back into the Fusion 360 software and underwent a mesh reduction process (a decrease of approximately 75% in the number of facets and vertices) to optimize the model for efficient printing and handling while preserving its anatomical integrity. This reduction step was crucial for ensuring printability and manageability without compromising accuracy. It was then manufactured using vat photopolymerization (Form 3+, Formlabs, Somerville, MA, U.S.A.) with Formlabs Elastic 50A Resin (Formlabs, Somerville, MA, U.S.A.). This method permitted the manufacturing of a phantom with a *Z* resolution (minimum layer height) of 0.025 mm and an *XY* resolution of 0.050 mm. The final product is visualized in Fig. 2F, G.

### Mechanical testing of fossa ovalis

The tensile properties of five different silicone mixtures were evaluated using an Instron Universal Testing Machine (Model 1011, Instron, Norwood, MA, U.S.A.) according to ASTM D638 standards [27]. The mixtures tested included EcoFlex$^{TM}$ 00-30 (EF30), EcoFlex$^{TM}$ 00-50 (EF50), and combinations with 25% and 50% slacker additives (SLK25 and SLK50). These materials were selected for their tissue-mimicking properties, which make them suitable for low-cost, functional 3D cardiovascular models [28, 29].

**Fig. 3:**
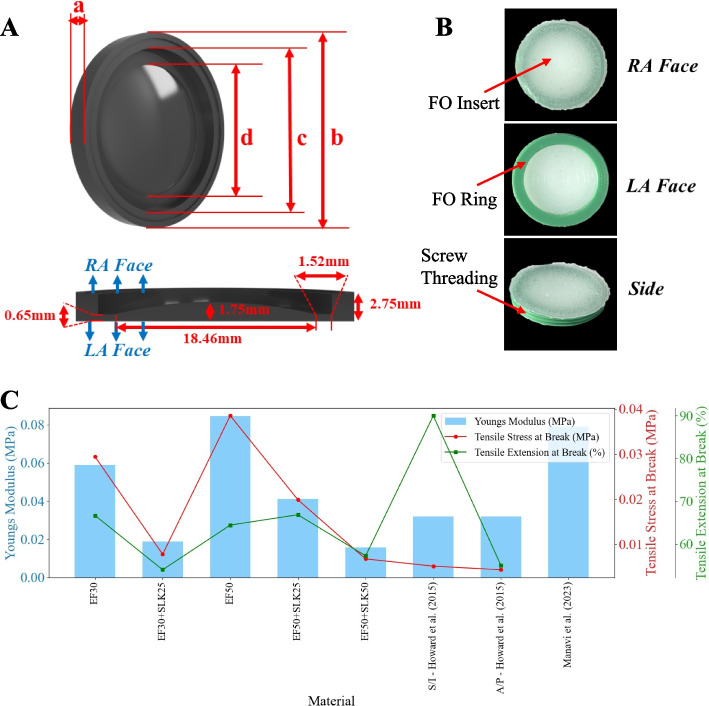
FO modeling and characterization. **A** Silicone mold of the FO based on patient-specific MRI segmentation, showing four key measurements: (a) maximum FO thickness, (b) FO diameter, (c) diameter of the interchangeable FO clamp, and (d) diameter of the molded silicone FO. **B** The resulting silicone FO insert after casting. **C** Comparison of mechanical properties of various silicone mixtures considered for FO development, including the mean Young’s modulus, mean tensile stress at break, and mean tensile extension at break. Abbreviations: MRI (magnetic resonance imaging); FO (fossa ovalis); EF (EcoFlex); SLK (slacker): S (superior); I (inferior); A (anterior); P (posterior)

Three samples were prepared and subjected to tensile testing for each silicone mixture, with three trial measurements performed on each sample until rupture. Figure 3C presents the mean tensile modulus, mean tensile stress for each silicone mixture at break, and mean tensile extension at break for the evaluated silicone mixtures. The tensile modulus provides insight into the material’s response to forces applied during tool positioning and needle puncture. In contrast, the tensile stress at break corresponds to the maximum force the material can withstand before rupture. The tensile extension at break indicates the material’s elasticity and flexibility, which are crucial for replicating the tenting behavior of the FO material.

The EcoFlex 00-50 silicone rubber, combined with a 50% slacker additive, was identified as the most suitable material for the FO insert, exhibiting a Young’s modulus of 0.016 MPa, a tensile stress at break of 0.007 MPa, and a tensile extension at break of 57.2%. These biomechanical properties closely resemble the characteristics of the human FO reported in the literature [20, 30].

In a comparative study, Howard et al. conducted mechanical testing on human FO tissues to determine their tensile properties using a standardized protocol [11]. The authors measured the normalized peak force, strain at failure, and Young’s modulus of the FO tissue. We approximated the average area reported in the authors’ paper to be 257 mm$^{2}$, thus translating the normalized peak forces into tensile stress at break for the two orientations, which are plotted in Fig. 3C. The Young’s modulus was consistently around 0.032 MPa for both orientations - 1.5 times less than that reported by Manavi et al. [20]. This highlights the variability in human physiological properties, which can be encapsulated by creating a replaceable FO capable of patient-specific customization in the future.

The Young’s modulus of the EF50+SLK50 (presented in Fig. 3C) mixture (0.016 MPa) closely matches the value reported for human FO tissue (0.032 MPa) by Howard et al., indicating that the selected material effectively mimics the flexibility and stiffness of human tissue.

However, it is important to note that the tensile stress at break for the EF50+SLK50 mixture (0.0068 MPa) is slightly greater than the normalized peak force values reported by Howard et al. for the superior/inferior (0.0052 MPa) and anterior/posterior (0.0044 MPa) orientations. This suggests that while the selected silicone mixture can withstand slightly greater forces before rupturing compared to human tissue, it still reasonably approximates the FO’s mechanical behavior.

#### Development of a replaceable fossa ovalis

The FO is a critical anatomical structure within the true IAS that is situated in the lower posterior region and separates the left and right atria. This thin fibrous tissue typically presents an oval or round depression and serves as the sole region through which the TSP kit can traverse to access the LA without entering the extracardiac space [30]. Accurate replication of the properties of the FO is crucial for developing a realistic TSP simulator.

Analysis of the patient’s IAS anatomy revealed no anatomical variations, such as a thick/fibrotic septum (defined as $\ge$3.0 mm at the TSP site [31]) or an atrial septal aneurysm. As shown in Fig. 3A, a silicone mold developed via material extrusion, was created with dimensions matching the patient’s specific FO anatomy and those reported in the literature [30].

Based on the results in Fig. 3C, EcoFlex 00-50 with 50% slacker (EF50+SLK50) was selected for the FO insert. The curved mold in Fig. 3A was filled with the EF50+SLK50 silicone mixture and cured, resulting in the FO depicted in Fig. 3B. The cured FO measured 18.46 mm in diameter with a thickness ranging from 0.65 mm to 1.75 mm. These dimensions correspond to approximately -0.11 to 3.96 standard deviations from the mean value reported by Howard et al. [11] and align with patient physiological properties (thickness, diameter, and tensile modulus) specified in [12, 20, 30, 32].

The present study employs a more robust material formulation compared to previous work by Thompson et al. [15], which used a harder silicone rubber (Dragon Skin 10 Medium, Smooth-On Inc., Macungie, PA, U.S.A.) for the FO. This choice was made to better mimic the tissue properties (particularly Young’s modulus) reported in [11].

**Fig. 4:**
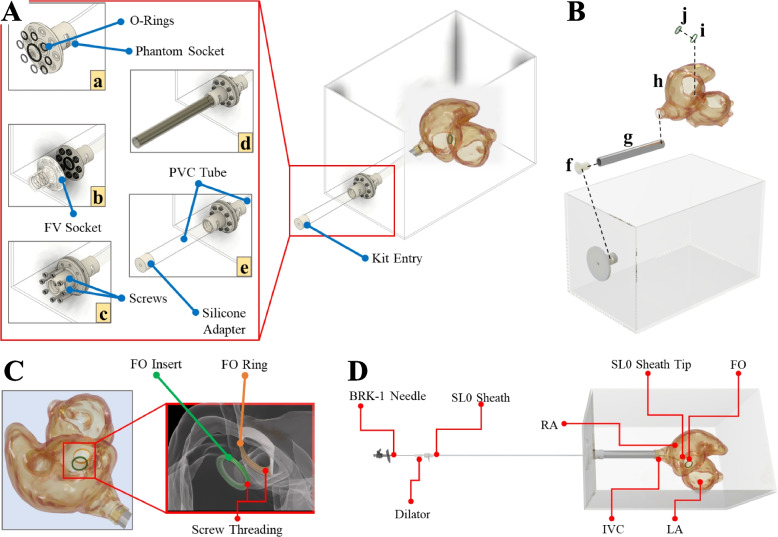
Simulator assembly. **A** Watertight assembly: The phantom was enclosed in a transparent box that facilitates X-ray fluoroscopy and ultrasound imaging by filling the box with water. (a) A PVC tube was attached from the phantom to a socket that was (b) secured to the box with (c) screws and O-rings placed on either side of the box and the (d) femoral vein socket and corresponding PVC tube, which was closed off with a (e) tear-resistant silicone adapter. **B** Phantom Assembly: (f) socket adapter and (g) PVC tube secured to the (h) phantom fitted with a permanent (i) FO ring and (j) replaceable FO insert. **C** FO attachment. **D** Complete assembly. Abbreviations: PVC (polyvinyl chloride); FV (femoral vein); FO (fossa ovalis); BRK (Brockenbrough); RA (right atrium); LA (left atrium); IVC (inferior vena cava)

The ring visualized in Fig. 3B was material extruded in Tough polylatic acid (PLA) (Polymaker Inc., Changshu, Jiangsu, China) to facilitate stable mounting and allow multiple punctures without replacing the entire phantom. The FO insert was then combined with the ring using cyanoacrylate adhesive (Fig. 4B). The FO insert and ring were threaded to enable quick FO replacement within seconds (Figs. 3B and 4C).

#### Simulator assembly

A polyvinyl chloride (PVC) tube connected the phantom to a box (serving as a water tank), simulating the femoral vein and IVC for tool insertion (Fig. 4D). A seal was secured to the PVC tube, acting as the entry point of the box (femoral vein) to maintain a watertight seal around the inserted tools while allowing tool manipulation during the procedure (Fig. 4A(e)). The water-filled box enabled imaging of the phantom with ultrasound. The adapter was cast in Mold Star$^{TM}$ 31T silicone (Smooth-On Inc., Macungie, PA, U.S.A.) for its tear-resistant properties, thus allowing smooth TSP kit manipulation while preventing the outflow of water during TSP simulation.

### Simulator evaluation

#### Pilot study setup

**Fig. 5:**
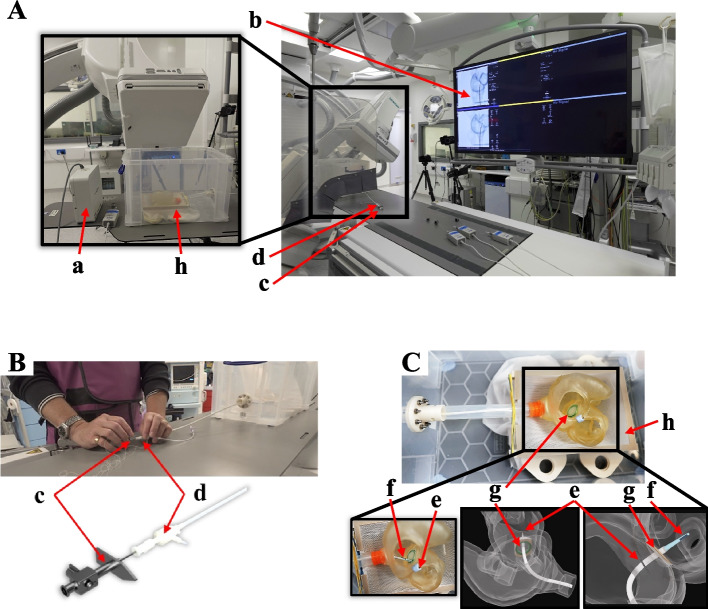
Experimental setup for the pilot study. **A** Adult catheterization laboratory, where the tools were tracked using (a) an Aurora EM-Field Generator and guided by (b) real-time X-ray fluoroscopy. **B** A cardiologist performing TSP using a (c) BRK-1 needle and (d) dilator-sheath. **C** Phantom fitted with (e) a reference sensor fastened to the cardiac structure above the (g) FO insert and a (f) sensor attached to the dilator tip. The phantom sits on a (h) mesh as the chest cavity. Abbreviations: EM (electromagnetic); TSP (transseptal puncture); BRK (Brockenbrough); FO (fossa ovalis)

Figure 5A depicts the experimental setup in the catheterization laboratory. One cardiology trainee and four cardiologists with 10, 10, 14, and 25 years of experience participated in the study. The TSP kit consisted of a standard 8.5 French transseptal sheath (SL0, St Jude Medical Inc. St Paul, MN, U.S.A.), a stiff dilator (curvature 270$^{\circ }$) (Torflex Superstrong, Baylis Medical Company, Inc. Montreal, Canada), and a BRK-1 needle (St Jude Medical Inc., St Paul, MN, U.S.A.) with a 53$^{\circ }$ curved distal end (Fig. 5B). Real-time X-ray fluoroscopy imaging was provided by an Artis Q biplane system (Siemens Healthcare GmbH, Forchheim, Germany).

The study’s primary objective (summarized in Fig. 1B) was to analyze the positioning of the puncture points on the FO using the global isotropy index (GII) and distance from the FO center. These metrics were chosen based on their clinical relevance and ability to quantify TSP performance [24, 33]. A root cause analysis also identified operator experience and puncture positioning as critical determinants of TSP success [9, 34]. Procedural time was recorded from when the TSP kit was withdrawn from the SVC until the needle crossed the septum. An Aurora$^{TM}$ electromagnetic (EM) tracking system was used to localize the tool’s tip position relative to the phantom (Fig. 5C). Participants performed one practice puncture, followed by 3-5 TSP attempts, puncturing the FO without a specific target (wherever they deemed best based on their experience). Only a single TSP was performed for each procedure, and the FO insert was replaced after each puncture.

#### Dimensional accuracy

The shape accuracy of the fabricated phantom was evaluated by comparing its dimensions to those of the STL model. Three independent researchers performed 50 measurements each to analyze the dimensional fidelity, taking physical measurements of the manufactured phantom using Vernier calipers (precision, ± 0.01 mm) and digital measurements of the STL model at the same points (Fusion360, Autodesk, San Francisco, CA, U.S.A.) (precision, ± 0.01 mm). These measurements were taken to cover key dimensions, such as the thickness of the atrial walls and the overall dimensions of the atrial chambers, IVC, and SVC. The results were then visualized on a Bland-Altman plot to compare the differences in the measurements taken from the physical phantom to those taken from the STL model.

#### Imaging compatibility

For the evaluation of imaging capabilities, a detailed comparison was conducted between the fluoroscopic and ultrasound images of the fabricated phantom and those obtained from real patients diagnosed with AF. The phantom ultrasound scan was obtained using a Philips EPIQ 7 ultrasound scanner (Philips, Amsterdam, The Netherlands) and an X6-1 3D probe in the bicaval view. In contrast, the real ultrasound scan was acquired using an X7-2t TOE probe with a Philips iE33 scanner (Philips, Amsterdam, The Netherlands) in the midesophageal bicaval and short-axis views during a Philips EchoNav Study at St Thomas’ Hospital. The fluoroscopic images were assessed for clarity in visualizing key procedural steps such as the positioning of the TSP kit, the tenting of the FO, and the needle puncture process. Similarly, the ultrasound images were examined to verify the phantom’s ability to reproduce essential anatomical structures, including the IAS and the FO, which are crucial for accurate needle positioning during TSP. The comparison was aimed at ensuring that the phantom could realistically replicate the visual and procedural guidance provided by these imaging modalities in clinical settings.

#### Global isotropy index

The global isotropy index (GII) is a quantitative measure adopted by Jayender et al. to quantify the dexterity of the catheter within the LA [24]. The authors leveraged this in an optimization algorithm to maximize the catheter reachability by finding the puncture location that maximized the GII score.

We employed this in our work to evaluate the patient-specific optimal TSP location by maximizing the maneuverability of a Thermocool SmartTouch Catheter (Biosense Webster Inc., CA, U.S.A.) within the LA [24]. Unlike Jayender et al., who utilized the Frenet-Seret model, we employed the constant curvature model to represent the catheter kinematics originating from the TSP site, around which the catheter pivots. The catheter model was constrained to access points within the LA in simple configurations, avoiding complex maneuvers such as looping the catheter on itself.

To determine the GII, the FO mesh was first exported from the CAD model. Each point on this FO mesh was considered a potential TSP site. The LA was then represented by a set of discrete points. The Jacobian matrix *J*, which describes the relationship between the catheter’s movement at the FO point and its corresponding positions within the LA, was calculated for each FO point.

The Jacobian matrix *J* can be expressed as:

1 $$\begin{aligned} J = \frac{\partial \textbf{x}}{\partial \textbf{u}}, \end{aligned}$$

where **x** represents the positions in the LA, and **u** represents the control inputs at the FO.

The GII at each FO point was computed as the ratio of the minimum to the maximum singular values of the Jacobian matrix *J*:

2 $$\begin{aligned} GII = \frac{\sigma _{min}(J)}{\sigma _{max}(J)}, \end{aligned}$$

where $\sigma _{min}(J)$ and $\sigma _{max}(J)$ are the minimum and maximum singular values of *J*, respectively. This ratio quantifies the isotropy of the catheter’s maneuverability: higher GII values indicate more uniform and flexible maneuverability of the catheter in all directions within the LA. The optimal TSP location was determined by finding the coordinates on the FO that maximized the GII, indicating the highest catheter maneuverability within the LA.

#### Questionnaire

A questionnaire comprising 6 closed-scale questions was designed alongside an expert cardiologist (who did not participate in the pilot study) to assess the clinicians’ perceptions of the performance and realism of the fabricated simulator following the simulated TSP procedure. The questions addressed user experience, tactile feedback, procedural realism, anatomical fidelity, and overall utility as a training and pre-procedural planning tool. Participants provided 5-point Likert scale responses ranging from 1 (strongly disagree) to 5 (strongly agree). Qualitative data from the questionnaire responses were analyzed using thematic analysis to identify common themes and patterns in user feedback.

#### Statistical analysis

All statistical analyses were performed utilizing SciPy (SciPy 1.13.1, Python 3.11.5, SciPy.org, Austin, TX, U.S.A.) [35]. A Bland-Altman analysis was conducted to assess the agreement between the dimensions of the STL model and the fabricated phantom. The Kruskal-Wallis test was used to evaluate differences in phantom measurements between the researchers who recorded the measurements. The Pearson correlation coefficient was used to assess the relationships between the GII score of the puncture points, distance from the FO centroid, and procedural time. The questionnaire responses were summarized using descriptive statistics, including the mean and standard deviation. A p-value $< 0.05$ was considered statistically significant for all analyses.

## Results

### Dimensional accuracy of biatrial structure

**Fig. 6:**
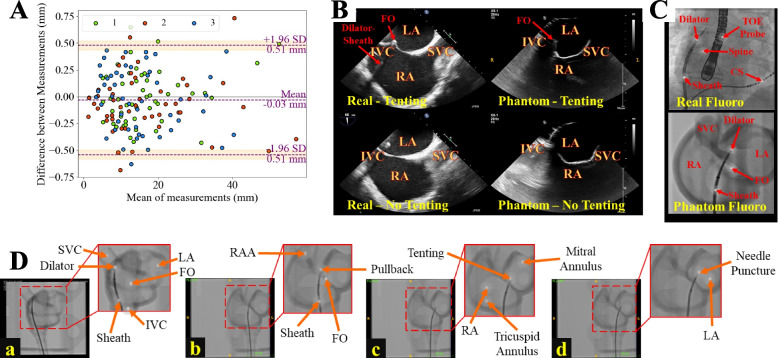
Imaging compatibility and shape accuracy evaluation of the patient-specific phantom. **A** Bland-Altman plot assessing the agreement between the dimensions of the STL model and the fabricated phantom. **B** Ultrasound imaging of the phantom, demonstrating its visualization of key anatomical structures, such as the FO, is essential for accurate TSP performance. Real ultrasound scans were obtained in the TOE bicaval view. **C** Real and phantom X-ray fluoroscopy images. **D** Real-time fluoroscopic guidance depicting critical steps in the TSP procedure, including (a, b) positioning of the TSP kit in the SVC in the RAO 30$^{\circ }$ and LAO 60$^{\circ }$ projections, respectively, (c) tenting of the fossa ovalis (FO), and (d) needle puncture. Abbreviations: STL (stereolithography); TSP (transseptal puncture); FO (fossa ovalis); SVC (superior vena cava); IVC (inferior vena cava); RAO (right anterior oblique); LAO (left anterior oblique); RA (right atrium); LA (left atrium); RAA (right atrial appendage); CS (coronary sinus)

A Bland-Altman plot (Fig. 6A) was generated to quantitatively assess the agreement (in the RA, LA, and IAS structures), with 96% of the data points falling within the 95% confidence interval (±1.96 SD). This close agreement underscores the accuracy and fidelity of the manufacturing process in replicating the patient-specific anatomy modeled in CAD. The mean difference between the STL file and the fabricated phantom dimensions was 0.03 mm (the 3D-printed phantom was smaller, on average, than the STL file), indicating minimal systematic bias in the manufacturing process. Statistical analysis using the Kruskal-Wallis test revealed no significant differences in measurements between the researchers ($p=0.38$), which further supports the reliability and consistency of the phantom’s dimensional accuracy with respect to the STL model.

### Imaging capability

The imaging compatibility of the patient-specific phantom was evaluated by comparing the X-ray fluoroscopy and ultrasound images of the phantom with those of real AF patients (different patients from the one used for the phantom). It is important to acknowledge that while the phantom was not specifically designed for full compatibility with imaging modalities such as X-ray fluoroscopy and ultrasound, it can be effectively utilized in conjunction with these modalities to guide and assess TSP performance.

The ultrasound images of the phantom (Fig. 6B) demonstrate the feasibility of using the phantom to guide TSP under ultrasound guidance despite the material properties not being optimized explicitly for ultrasound compatibility. The phantom ultrasound images allow the visualization of critical anatomical structures, such as the FO and IAS, which are essential for accurate needle positioning. The real ultrasound scan obtained using 2D TOE in the bicaval view, provides a more detailed and comprehensive visualization of the cardiac anatomy, showcasing the FO, IAS, and surrounding structures with greater clarity than the phantom images.

The X-ray fluoroscopy images of the phantom were acquired in air (not in a water-filled tank), which may exhibit variation in appearance when submerged in water. The phantom image in Fig. 6C(lower) demonstrates clear visualization of the biatrial shell, FO during tenting, and TSP kit. However, the real fluoroscopic image (Fig. 6C(upper)) includes additional landmarking tools commonly used by cardiologists during TSP, such as a TOE probe and a catheter placed in the coronary sinus (CS), which are not present in the phantom image. The CS catheter delineates the margin of the left atrial free wall, while a diagnostic catheter at the Bundle of His (not shown in the figure) is used to identify the anterior aspect of the IAS. The spine is also used as a landmark for guiding the orientation of the TSP kit in the RAO 30$^{\circ }$ X-ray fluoroscopy projection[5]. Furthermore, the cardiac structures of the phantom images are more visible (in higher contrast) than those in real patient X-ray fluoroscopy images.

Figure 6D illustrates the real-time X-ray fluoroscopic guidance during the simulated TSP procedure, depicting key steps such as parking the TSP kit in the SVC (Fig. 6D(a)), kit pullback (Fig. 6D(b)), tenting of the FO (Fig. 6D(c)), and needle puncture (Fig. 6D(d)).

### Puncture point analysis

**Fig. 7:**
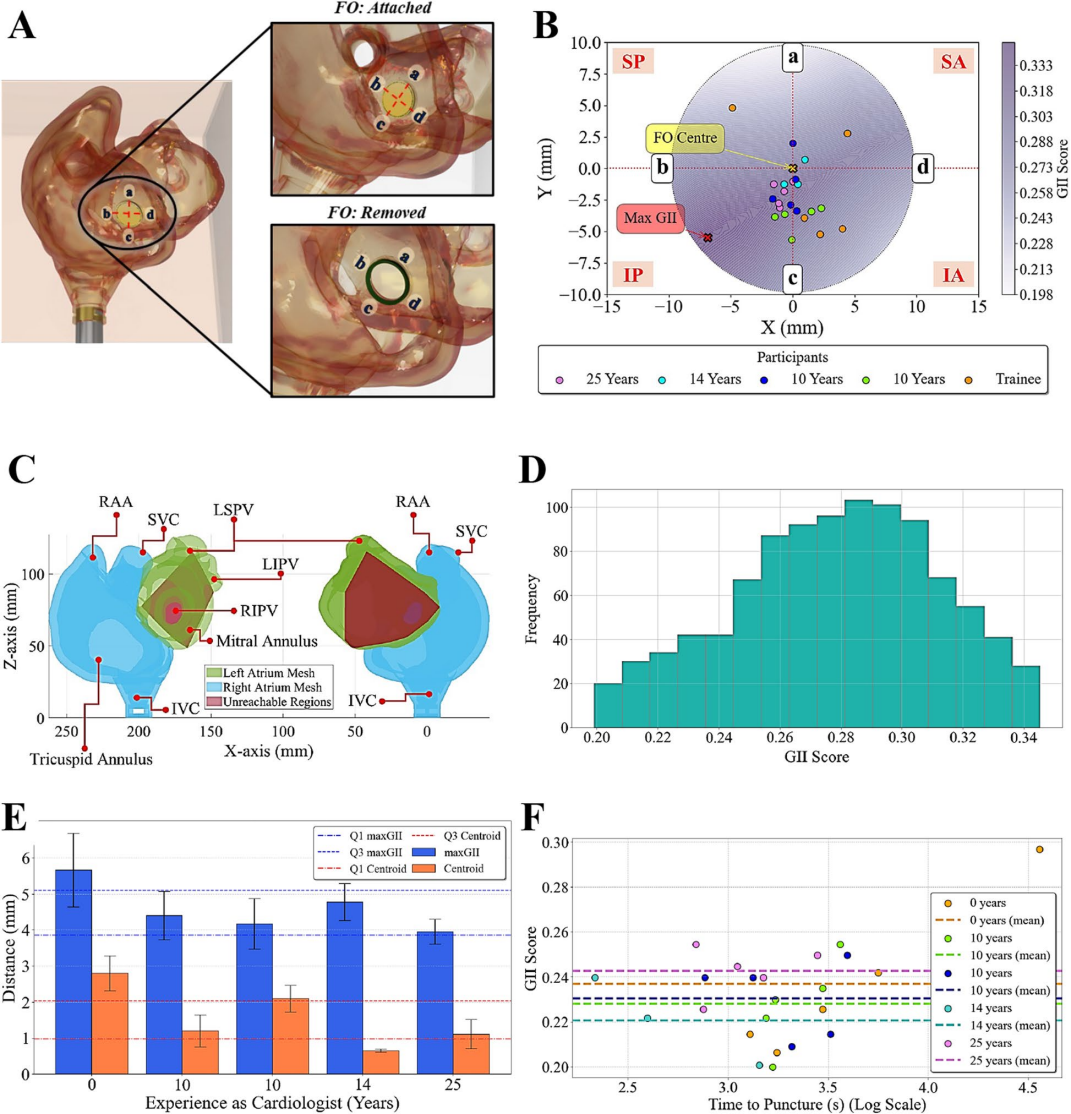
Puncture positioning. **A** Orientation of the FO and its division: (a), (b), (c), and (d) correspond to superior, posterior, inferior, and anterior orientations of the FO based on the schematic divisions (SP, IP, SA, and IA) defined in [9]. **B** GII scores and participant puncture locations on the FO mesh. **C** Regions on the LA unreachable by the catheter, given the catheter pivots about the patient-specific optimal puncture location. D Histogram of the frequency of GIIs. **D** GII scores against the time taken to perform TSP. **E** Mean Euclidean distance of participants’ puncture locations from the maximum GII score and FO centroid coordinates. **F** GII scores against the logarithmic scale of time. Abbreviations: FO (fossa ovalis); SP (superior-posterior); IP (inferior-posterior); SA (superior-anterior); IA (inferior-anterior); GII (global isotropy index)

The puncture points achieved by each participant during the simulation sessions were tracked using the EM tracking system and recorded relative to a reference sensor placed on the biatrial structure (Fig. 5C). Figure 7A illustrates the orientation of the FO and the division into superior (S), posterior (P), inferior (I), and anterior (A) regions based on the schematic divisions (SP, IP, SA, and IA) defined by [9].

The results of the GII optimization algorithm are visualized in Fig. 7B, which presents a set of discrete points on the FO mesh representing the GII values that correlate with the dexterity of the catheter in the LA. The orientation of the 2D FO is given with respect to the 3D anatomical structure in Fig. 7A. The heatmap visualizes how a change in puncture positioning can affect a catheter’s isotropy, with higher scores indicating improved catheter maneuverability within the LA. A higher index score can be achieved around the inferoposterior region of the FO, closer to the IVC, thus providing the greatest range of catheter motion for pulmonary vein isolation - similar to conclusions drawn about the advised puncture region for LA catheter ablation in real procedures [4, 36]. The participant puncture points across 3-5 trials are overlaid on the FO mesh. The data points from experienced cardiologists clustered closer to the regions of the FO with the highest GII, indicating a relationship between experience and puncture point accuracy.

The patient-specific optimal puncture point was defined on the FO based on the location of the coordinates of the maximum GII. The LA was then overlaid with regions unreachable by the catheter (in red) when pivoted about the maximum GII location, as shown in Fig. 7C, highlighting the importance of selecting the optimal puncture site for maximizing catheter maneuverability. The posterior wall of the LA, as well as the RSPV and RIPV, was unreachable by the catheter. The right-sided PVs are potentially challenging to access during pulmonary vein isolation, sometimes requiring a steerable sheath to provide the catheter with increased maneuverability [37].

The histogram in Fig. 7D illustrates the frequency distribution of GII scores across the FO mesh. This finding revealed that a significant portion of the FO yielded suboptimal catheter maneuverability within the LA for this patient’s specific anatomy, as evidenced by the high frequency of low GII scores, with 0.29 being the most common score.

Figure 7E analyses the relationship between the participants’ experience as cardiologists and their performance in selecting puncture sites, as measured by the distance from the maximum GII coordinates and the FO centroid. The results demonstrated a clear trend: the trainee exhibited the greatest mean distance from the maximum GII and the FO centroid compared to the more experienced cardiologists. This finding suggests that less experienced participants choose puncture sites farther from the ‘optimal location’, as determined by the maximum GII and the FO centroid. The increased variability in the spread of the puncture points achieved by the trainee highlights the inconsistency in their puncture site selection criteria.

In contrast, the findings also imply that more experienced cardiologists select puncture sites that are consistently closer to both the maximum GII and the FO centroid (in the inferoposterior region of the FO of this specific patient). All the participants chose points closer to the FO centroid - which, in our work, is the point at which the FO is thinnest - than to the coordinates of the maximum GII score. The absence of a significant difference between the two metrics within these experienced participants suggests a more consistent and optimal approach to puncture site selection.

Figure 7F depicts the relationship between GII scores and procedural time, revealing a strong positive correlation (Pearson’s $r = 0.656$, $p < 0.05$). This suggests that as the GII increases, the time taken to perform TSP also tends to increase; puncture points with higher GII scores, which correspond to better catheter maneuverability within the LA, are associated with longer procedural times. It is possible that operators may take more time to carefully position the catheter at these optimal puncture locations to ensure better outcomes. The Pearson correlation coefficient between centroid distance and procedural time ($r = 0.566$, $p < 0.05$) indicates that as the distance from the centroid increases, the time taken to perform TSP also tends to increase. This finding suggests that puncture points farther from the center of the FO may require more time for the operator to navigate and position the catheter accurately. However, the correlation coefficient between GII and centroid distance ($r = 0.314$, $p > 0.05$) indicated no significant relationship between the GII score and the distance from the centroid.

These results highlight the complexity of factors influencing TSP procedural time and emphasize the importance of considering both the GII and centroid distance when selecting optimal puncture points. While higher GII scores are associated with better catheter maneuverability, they may not always translate to faster procedural times. Similarly, puncture points closer to the centroid may not necessarily have higher GII scores. These findings underscore the importance of considering multiple factors when selecting optimal puncture points and highlight the need for further research to better understand the complex relationships between these variables in TSP procedures.

### Questionnaire analysis

The responses to the questionnaire provided information on the participants’ perceptions of the phantom’s realism, utility, and training potential. The mean scores for each question category across all the participants ($n=5$) are presented in Table 1. The phantom received high scores for providing a realistic anatomical representation (Q1, 4.15 ± 0.40) and allowing a moderately realistic visualization of the TSP site under fluoroscopic guidance (Q3, 3.75 ± 0.43). The participants found the tactile feedback and resistance of the phantom during TSP to be realistic (Q2, 4.50 ± 0.50), and the tactile sensations were considered reasonably representative of clinical procedures (Q4, 4.00 ± 0.50). Overall satisfaction with the phantom was favorable (Q5, 4.00 ± 0.50), and participants rated the challenge of training on the simulator as straightforward (Q6, 4.60 ± 0.40). The open-ended feedback provided additional insight into the phantom’s strengths and areas for improvement. The participants commended the realistic anatomical representation and the ability to visualize procedural steps, such as tenting of the FO, which is challenging to observe during actual TSP. However, some have suggested the use of exchangeable FO inserts with varying flexibility and thicknesses to incorporate the difficulties associated with TSP during repeat catheterization into the training simulator.

**Table 1:** Face validation of the 3D-printed patient-specific model in clinical circumstances

No.	Question Statement	Mean $\varvec{\pm }$ SD^a^
Q1	The phantom provides a realistic anatomical representation	4.15 ± 0.40
Q2	The phantom accurately simulates the tactile feedback and resistance encountered during a real TSP procedure	4.50 ± 0.50
Q3	Under X-ray fluoroscopy guidance, the phantom allows for realistic visualization of the TSP site and surrounding structures	3.75 ± 0.43
Q4	The phantom’s textures provide the sensory realism experience in clinical procedures	4.00 ± 0.50
Q5	Rate your overall satisfaction with the phantom	4.00 ± 0.50
Q6	How difficult would you rate the challenge of training on the simulator^b^	4.60 ± 0.40

The scores, ranging from 1 (strong disagreement) to 5 (strong agreement), were provided by the participants following their final trial. The responses are presented as the mean agreement ± SD for all the participants (n=5). Abbreviations: SD (standard deviation); TSP (transseptal puncture)

^a^1=strongly disagree, 5=strongly agree

^b^1=extremely challenging, 5=extremely straightforward

## Discussion

The present study introduces a novel TSP simulator that integrates patient-specific anatomical accuracy, imaging capabilities, and quantitative performance metrics to enhance cardiology training. This study aimed to evaluate the simulator’s accuracy, with a particular focus on its suitability for procedural simulation. The simulator’s design prioritizes high-fidelity reproduction of cardiac anatomy, particularly the FO, to provide a realistic tactile experience during contact of the TSP kit with the IAS and FO, as well as needle puncture. The successful evaluation of the simulator within a clinical setting involved a diverse group of cardiologists and a trainee, which demonstrated its potential for integration into existing training workflows and its ability to improve procedural skills in a safe, controlled environment.

A key strength of our simulator lies in its ability to be imaged using both X-ray fluoroscopy and ultrasound modalities, distinguishing it from other TSP simulators [6, 14, 15, 18, 19]. The fluoroscopic images demonstrated that the phantom’s design allows for clear visualization of the TSP procedure, including the positioning of the TSP kit, tenting of the FO, and needle puncture. This visualization capability is crucial for providing real-time guidance and feedback to the operator during the simulated procedure. This versatility enhances the simulator’s utility and applicability in diverse training environments, ensuring that trainees are well-prepared to perform TSP under the different imaging conditions encountered in practice. Furthermore, this imaging capability paves the way for the potential use of the simulator in medical device testing and the evaluation of robotic TSP systems [38].

The incorporation of a replaceable FO component in our simulator improves its cost-effectiveness and sustainability in clinical training programs, while the integration of the GII introduces a novel quantitative metric for assessing TSP performance [24]. By providing detailed feedback on catheter maneuverability and puncture location, the GII facilitates a deeper understanding of the choice of puncture position involved in TSP for varying left-sided cardiac interventions, enabling trainees to refine their technique and improve procedural accuracy [4]. This quantitative assessment adds a layer of objective evaluation not typically provided in other simulators, offering a more comprehensive and evidence-based approach to TSP training [24].

The variability observed in puncture point positioning among participants underscores the importance of ongoing training and experience in achieving consistent and precise TSP performance. Experienced cardiologists tended to cluster their puncture points closer to regions of the FO associated with higher GII scores, indicating a relationship between experience and optimal puncture localization. Conversely, the puncture points of the least experienced participant exhibited more significant variability, highlighting the potential role of simulation-based training in shortening the learning curve associated with this critical skill.

The questionnaire analysis provided insights into participants’ perceptions of the phantom’s realism, utility, and training potential. While participants generally agreed on the phantom’s realistic anatomical representation and accurate visualization of the TSP site under X-ray fluoroscopic guidance, the variability in responses suggests areas for potential improvements, such as refining tissue properties to better mimic the tactile sensations encountered during TSP.

The results achieved with our simulator align with emerging trends in simulation-based medical education, emphasizing personalized learning experiences [3, 22]. Adapting the simulator to individual patient anatomy through patient-specific modeling and providing quantitative feedback via the GII allows for tailored training experiences that cater to each trainee’s specific needs and skill levels. This personalized approach to TSP training is particularly relevant given the anatomical variations encountered in clinical practice, underscoring the importance of simulators that can accommodate diverse patient scenarios for optimal procedural planning and execution.

### Limitations

The limitations of this study should be acknowledged. The absence of certain landmarking tools (shown in Fig. 6C(upper)) in the phantom fluoroscopic image and the differences in image quality and anatomical detail between the real and phantom ultrasound scans highlight areas for potential improvement in future iterations of the phantom design. These include a TOE probe, a CS catheter, a diagnostic catheter in the Bundle of His, and the spine. The differences in image quality between the real and phantom ultrasound scans can be attributed to several factors. Firstly, the real ultrasound scan was obtained using a TOE probe, which provides superior image quality and anatomical detail compared to the phantom scan obtained using a surface probe. Secondly, the material properties of the phantom, while suitable for demonstrating the basic anatomy and guiding TSP, do not fully replicate the acoustic properties of human tissue, resulting in some discrepancies in image quality and contrast. Enhancing the phantom’s material properties to better mimic the acoustic properties of human tissue and incorporating these additional anatomical landmarks could further improve its fidelity to real-world clinical scenarios [39]. Additionally, integrating TOE and ICE imaging into these training modules remains an area for future development.

The relatively small sample size of participants may limit the generalizability of the findings. Future studies should aim to recruit a larger cohort of cardiologists and trainees to further validate the effectiveness of the simulator and identify potential areas for improvement. Additionally, while the simulator’s performance was evaluated in a clinical setting, long-term follow-up studies are necessary to assess its impact on procedural success rates and patient outcomes, as was done in [3].

### Future work

Future work should focus on further enhancing the simulator’s imaging capabilities and fidelity to real-world clinical scenarios. The integration of TOE and ICE imaging into the training modules would provide trainees with a more comprehensive experience. Moreover, incorporating additional anatomical landmarks and refining the phantom’s material properties to better mimic the acoustic properties of human tissue would further improve the simulator’s realism. Conducting studies with larger sample sizes and long-term follow-up assessments would help establish the simulator’s impact on procedural success rates and patient outcomes, validating its effectiveness as a training tool. Lastly, exploring the potential applications of the simulator in medical device testing and robotic TSP system evaluation could open up new avenues for research and innovation in the field of cardiology.

## Conclusion

The TSP simulator demonstrated potential for being incorporated into electrophysiology/cardiology training programs to provide trainees with an opportunity to learn tool handling and improve spatial coordination and manual dexterity prior to performing the procedure on real humans. By integrating patient-specific anatomical accuracy, imaging capabilities, and quantitative performance metrics, the simulator offers a comprehensive and adaptable training platform that can enhance procedural skills and patient safety. The successful evaluation of the simulator in a clinical setting highlights its potential for real-world application and integration into existing training programs. As the dependency on TSP grows with the increasing prevalence of AF cases, the development and refinement of high-fidelity simulators, such as the one presented in this study, will play an increasingly crucial role in preparing practitioners to perform complex procedures with confidence and precision.

## Data Availability

The dataset generated and analysed during the current study are available in the GitHub repository, https://github.com/ayaziee/Biatrial-Phantom-for-Transseptal-Puncture-Training.

## Abbreviations

TSP: Transseptal puncture
AF: Atrial fibrillation
FO: Fossa ovalis
LA: Left atrium
IAS: Interatrial septum
TOE: Transesophageal echocardiography
ICE: Intracardiac echocardiography
AM: Additive manufacturing
MJ: Material jetting
FDM: Fused deposition modeling
GII: Global isotropy index
STL: Standard tessellation language
SLA: Stereolithography
RA: Right atrium
MRI: Magnetic resonance imaging
IVC: Inferior vena cava
SVC: Superior vena cava
CAD: Computer-aided design
RAA: Right atrial appendage
IVC: Inferior vena cava
RSPV: Right superior pulmonary vein
RIPV: Right inferior pulmonary vein
LSPV: Left superior vena cava
PLA: Polylactic acid
PVC: Polyvinyl chloride
EM: Electromagnetic tracking
CS: Coronary sinus
RAO: Right anterior oblique
LAO: Left anterior oblique
SP: Superior-posterior
IP: Inferior-posterior
SA: Superior-anterior
IA: Inferior-anterior
SD: Standard deviation
